# Dentate gyrus abnormalities in sudden unexplained death in infants: morphological marker of underlying brain vulnerability

**DOI:** 10.1007/s00401-014-1357-0

**Published:** 2014-11-25

**Authors:** Hannah C. Kinney, Jane B. Cryan, Robin L. Haynes, David S. Paterson, Elisabeth A. Haas, Othon J. Mena, Megan Minter, Kelley W. Journey, Felicia L. Trachtenberg, Richard D. Goldstein, Dawna D. Armstrong

**Affiliations:** 1 Department of Pathology, Boston Children’s Hospital and Harvard Medical School, Boston, MA USA; 2 San Diego County Medical Examiner’s Office, San Diego, CA USA; 3 New England Research Institutes, Watertown, MA USA; 4 Department of Pediatrics, Boston Children’s Hospital and Harvard Medical School, Boston, MA USA; 5 Department of Pathology (Emeritus), Texas Children’s Hospital and Baylor School of Medicine, Houston, TX USA

## Abstract

Sudden unexplained death in infants, including the sudden infant death syndrome, is likely due to heterogeneous causes that involve different intrinsic vulnerabilities and/or environmental factors. Neuropathologic research focuses upon the role of brain regions, particularly the brainstem, that regulate or modulate autonomic and respiratory control during sleep or transitions to waking. The hippocampus is a key component of the forebrain–limbic network that modulates autonomic/respiratory control via brainstem connections, but its role in sudden infant death has received little attention. We tested the hypothesis that a well-established marker of hippocampal pathology in temporal lobe epilepsy—focal granule cell bilamination in the dentate, a variant of granule cell dispersion—is associated with sudden unexplained death in infants. In a blinded study of hippocampal morphology in 153 infants with sudden and unexpected death autopsied in the San Diego County medical examiner’s office, deaths were classified as unexplained or explained based upon autopsy and scene investigation. Focal granule cell bilamination was present in 41.2 % (47/114) of the unexplained group compared to 7.7 % (3/39) of the explained (control) group (*p* < 0.001). It was associated with a cluster of other dentate developmental abnormalities that reflect defective neuronal proliferation, migration, and/or survival. Dentate lesions in a large subset of infants with sudden unexplained death may represent a developmental vulnerability that leads to autonomic/respiratory instability or autonomic seizures, and sleep-related death when the infants are challenged with homeostatic stressors. Importantly, these lesions can be recognized in microscopic sections prepared in current forensic practice. Future research is needed to determine the relationship between hippocampal and previously reported brainstem pathology in sudden infant death.

**Electronic supplementary material:**

The online version of this article (doi:10.1007/s00401-014-1357-0) contains supplementary material, which is available to authorized users.

## Introduction

The sudden infant death syndrome (SIDS) is the sudden and unexpected death of an infant less than 1 year of age that remains unexplained after a complete autopsy, death scene investigation, and review of the clinical history [60]. It is the leading cause of postneonatal infant mortality in the United States today with an overall incidence of 0.57/1,000 live births [38]. Typically it occurs in a sleep period, either during sleep itself or one of the many transitions between sleep and arousal during night sleep or naps [28]. Over the last two decades, a major focus of research into the pathogenesis of sudden, sleep-related, and unexplained death in infants has been upon the brain, with the over-arching hypothesis that brain systems which mediate autonomic and/or respiratory control during sleep in a critical developmental period are compromised [26, 28]. Historically, attention has focused upon the brainstem in SIDS research [15, 26, 28]. Abnormalities involving the neurotransmitter serotonin (5-HT) have been reported and confirmed in a subset of SIDS cases [9, 25, 26, 32, 40, 41, 44, 58]. In addition, the possibility that sudden unexplained death in infants is due to a lethal (unwitnessed) seizure has also been postulated [16, 50, 55]. In support of this idea, we recently reported on a previously healthy infant (separate from the cohort presented below) who was discovered seizing by his father during a sleep period, and whose autopsy was unrevealing [24]. Of major anatomic interest pertaining to the seizure hypothesis is the hippocampus which is a critical locus in the limbic forebrain network that modulates respiration, heart rate, blood pressure, and temperature according to behavioral state via its projections to brainstem sites [46, 52, 53, 61]. Moreover, it is prone to the generation and propagation of seizures [42, 48], and hippocampal lesions are associated with temporal lobe epilepsy and sudden unexplained death in epilepsy (SUDEP) [1, 2, 5, 17, 37]. Until recently, brain research in SIDS has emphasized only subtle hippocampal pathology, consistent with chronic hypoxia [6, 15, 19, 39, 59]. Our observation of dentate gyrus abnormalities in certain SIDS cases reminds us that the disorganization of hippocampal circuitry, either through developmental or acquired abnormalities, may predispose an individual to abnormal electrical activity that is propagated to the brainstem, leading to autonomic and/or respiratory disruption without tonic–clonic movements, i.e., autonomic seizures, and sudden death. We suggest that hippocampal disorganization in a predisposed infant may lead to cardiorespiratory instability (before the clinical onset of seizures) by functional impairment of the limbic–brainstem homeostatic network and death.

Our laboratory began to focus upon a potential role of the hippocampus and its interactions with the brainstem in sudden unexplained death in infants based upon our in-house observations of focal granule cell bilamination (FGCB), a variant of granule cell dispersion, in the dentate gyrus (DG) in two individual SIDS brains (not part of the cohort below) that were received over several months for diagnostic neuropathologic consultation. We also noted granule cell dispersion in a third SIDS infant (also not part of the cohort below) in association with mild, gross asymmetry of the hippocampus that we recently reported [51]. Granule cell dispersion, including FGCB, has been reported almost exclusively in the brains of patients with temporal lobe epilepsy, implicating it in hippocampal epileptogenesis [1, 2, 5, 17]. A seizure-related mechanism during sleep has been suspected in SIDS in part because sudden and unexplained death in epilepsy (SUDEP) and SIDS are both sudden, unexpected, and typically sleep-related death unexplained by autopsy [16, 50]. We have also reported hippocampal anomalies, including FGCB, in a subset of small children (1–6 years old) with sudden unexplained death in childhood (SUDC) with or without a personal and/or family history of febrile seizures [22, 23]. Because the diagnostic age criteria of SIDS is <1 year and SUDC is >1 year, some infants with dentate disorganization may possibly survive infancy to die suddenly as toddlers, possibly potentiated by febrile seizures. In the current study, we focused upon the hippocampus in sudden unexplained deaths in infants because of the potential for elucidating the role of seizures in such deaths, as well as a possible continuum of hippocampal/seizure-related deaths linked to sudden death across the age spectrum, including SUDC and SUDEP [27].

In the following study, we tested the hypothesis that the frequency of FGCB is significantly increased in the dentate gyrus in infants with sudden unexplained death compared to infants who died suddenly due to explained causes. Because autopsies of sudden unexpected death in infants—explained and unexplained—are under the legal jurisdiction of the medical examiner’s office in the United States, the study was necessarily performed utilizing the available archival neuropathological materials in a medical examiner’s system that typically included a single hippocampal section at a random level, and had limited capability for special tissue studies. The objective in this preliminary study was to determine if FGCB was detectable and increased in frequency in at least a single random hippocampal section of cases with sudden unexplained infant death that would warrant future mobilization of resources and technical support for a future in-depth, bilateral, and serial section examination of the hippocampus in the medical examiner’s system. In this initial study, we performed a blinded study of hippocampal morphology in tissue sections with the standard light microscope in a large cohort of infants with sudden death (both explained and unexplained, total *n* = 153) who were autopsied in the medical examiner’s office in San Diego County, CA, USA. We found that FGCB was significantly increased in frequency in the unexplained group compared to the explained group, with 41.2 % of the unexplained group affected. This major observation warrants, in our opinion, further in-depth analysis of the hippocampus in unexplained infant death.

## Materials and methods

### Tissue accrual

Microscopic sections of hippocampus (when available) were examined from infants autopsied at the San Diego County medical examiner’s office. All infants died suddenly and unexpectedly between 1991 and 2012. Tissues were available for research under the auspices of the California statute (SB 1067).

### Classification of death

We classified the infant deaths according to a scheme of unexplained versus explained by review of clinical history, complete autopsy, and death scene investigation that we developed. Unexplained deaths were those in which a complete autopsy, death scene investigation, and review of the clinical history, and circumstances of death, including cardiopulmonary resuscitation status, did not reveal a known cause of death. Explained deaths were those in which the postmortem studies revealed a known cause of death, including infection, accident, homicide, or unequivocal asphyxia, defined as documented obstruction of the nose and mouth, or chest constriction upon the death scene investigation. Infants with somatic and/or brain malformations, or known seizures disorders, were excluded. In all cases, information about multiple prenatal, neonatal, and postnatal parameters, including history of seizures, were collected from the clinical and autopsy records available. The explained cases served as the controls in this study.

Due to the debate about the potential role of hypoxia/asphyxia in sudden infant death related to unsafe sleep environments [33, 49, 54, 56], we further subdivided cases according to an “asphyxia risk profile”, modified from Randall et al. [49], and similar, but not identical, to that proposed recently by Shapiro-Mendoza et al. [54]. Explained deaths were subdivided into those with or without acute hypoxic insult as the immediate cause, the former including drowning and intentional suffocation. Unexplained deaths were subdivided into: (1) death occurring in the setting of recommended sleep practices, as delineated by the Task Force on Sudden Infant Death Syndrome [56]; (2) death occurring in the setting of unrecommended sleep practices [56]; and (3) possible suffocation (airway obstruction) by history, but lacking physical evidence on autopsy. A study investigator expert in SIDS (RDG) undertook case adjudications with the asphyxia risk profile blinded to the hippocampal data. The data in this manuscript are mainly reported using this adjudication system. In parallel analysis, we examined the hippocampal data with the classification of the infant deaths according to typical systems, as utilized by the San Diego County medical examiner’s office. In this system, SIDS was defined as the sudden and unexpected death of an infant, which remains unexplained after a complete autopsy, death scene investigation, and clinical review of the history [60]. Undetermined was defined as a sudden and unexpected infant death in which the cause of death was not established with certainty because of equivocal findings at autopsy and/or upon scene investigation, or findings of uncertain significance relative to lethality. The circumstances of death included the setting of unrecommended sleep practices at the time of death that may have, but not conclusively, resulted in unintentional asphyxia [60]. The following subcategories comprised the explained category: known natural causes of death, homicide, accidental trauma, and accidental suffocation. This classification was performed by consensus of the medical examiners, including the study member (OJM), in their daily staff meetings, and blinded to the hippocampal data.

### Hippocampal study review

Any macroscopic abnormalities of the brain were recorded from autopsy reports. The San Diego medical examiners had made coronal sections of the hippocampus at different (non-standardized) levels anterior to posterior. We identified the levels by adjacent landmarks in the same section, or by the level-specific configuration of the hippocampus [10, 20]. Given the well-recognized variation in cellular architecture of the human hippocampus throughout its structure, anterior (level of pes), body (level of lateral geniculate nucleus), and posterior (level of pulvinar), inclusion in the study required that the sections be oriented properly in the coronal plane, as identified by adjacent anatomic landmarks and in comparison to normative human atlases of the hippocampus [10, 20]. Cases without proper orientation of the dentate gyrus in the hippocampal section, or that were technically unsatisfactory (fold, tears), were excluded from analysis. The hippocampal sections, 6 μm thick, were stained with hematoxylin and eosin, and examined with the standard light microscope. Two pediatric neuropathologists (HCK and DDA) analyzed the hippocampal sections independently; cases with discordant observations were reviewed together to achieve consensus. The autopsy reports were reviewed for gross and microscopic abnormalities in brain regions outside of the hippocampus, and only cases with major malformations or clinical history of epilepsy or seizures were excluded.

### Histological features

We identified 44 developmental and acquired features in the DG, Ammon’s horn, subiculum, entorhinal cortex, temporal cortex and white matter (Supplementary Table 1). Because of the biologic plausibility of neurogenesis induced by hypoxia–ischemia (HI) [4, 29], we defined FGCB-HI with associated acute and chronic changes consistent with HI (e.g., neuronal hyper-eosinophilia and/or loss, gliosis), distinct from FGCB without HI changes. We hypothesized that FGCB-HI was due to adverse cardiorespiratory events prior to or around the time of death, and would be found in all categories. To characterize further immature-appearing cells in the subgranular layer of the DG, we performed standard immunocytochemistry using immunomarkers for reactive astrocytes (glial fibrillary acidic protein), activated microglia (CD68), and immature neurons (Tuj1) [35] in three representative cases (Table 1). We determined the frequency of microdysgenetic features in the temporal cortex and white matter in the hippocampal sections, i.e., columnar arrangement of neurons, neuronal cytomegaly, and neuronal clustering in the temporal cortex, and nodular heterotopia or single ectopic neurons in white matter (Supplementary Table 1).

**Table 1:** Immunocytochemical methods for analysis of immature-appearing cells in the dentate gyrus of the hippocampus

Antibody	Cell type identified	Company	Catalogue number	Source	Concentration	Antigen retrieval
GFAP	Astrocytes	Ventana Medical Systems, Tucson, AZ	760–4345	Rabbit	Predilute—ready to use	Ventana’s cell conditioner 1 (CC1) 8 min
CD68 (KP-1)	Reactive microglia	Ventana Medical Systems, Tucson, AZ	790–2931	Mouse	Predilute—ready to use	Ventana’s cell conditioner 1 (CC1) 30 min
Tuj1 (Beta III Tubulin)	Neuronal progenitors	Abcam, Cambridge, MA	14545	Mouse	1:5,000	Microwave 198 °F for 20 min citrate buffer, pH 6.0

Paraffin-embedded tissue was deparaffinized and rehydrated in a series of xylenes and alcohols of decreasing concentration. Specific antigen retrieval methods for each antibody are indicated. Antibody detection for GFAP and CD68 was performed with the Ventana’s ultraView Universal DAB Detection Kit (catalogue number 760-500, Ventana Medical Systems, Tucson, AZ, USA) according to manufacture’s instruction. Antibody detection for Tuj1 was performed with the Dako EnVision + System- HRP (DAB) (catalogue number K4006, Dako, Carpinteria, CA, USA) according to manufacture’s instruction

In pilot reviews of the infant hippocampus in tissue sections, we observed thick walled blood vessels not described in previous reports of human hippocampal development, and thus of uncertain significance. These vessels were small arteries and capillaries, with hypertrophic media and prominent endothelial nuclei; they were identified in the hilus, granule cell layer, and/or molecular layer of the DG (Supplementary Table 1). The presence of these vessels were recorded by us to determine if they were a normative feature of infant hippocampal development, associated with putative dentate gyral pathology in unexplained deaths in infants, or with hippocampal hypoxic–ischemic pathology as a possible angiogenic response.

### Statistical analysis

Characteristics were compared between subcategories using analysis of variance for age and postmortem interval (PMI) and *χ* ^2^ tests for sex, race, and prematurity. Hippocampal features were compared between groups using Fisher exact tests. Effects of age and PMI on each hippocampal feature were tested via logistic regression, controlling for case classification. For features with significant effects of age or PMI, we report comparison between groups adjusted for age/PMI via logistic regression. Associations between features were tested with Fisher exact tests of each hippocampal feature by presence/absence of FGCB. Tests for phenotype of FGCB included *t* tests for age and *χ* ^2^ tests for sex, race, prematurity, sleep position, sleep location, co-sleeping, and minor illness. All analyses were conducted using SAS v9.3 (SAS Institute Inc., Cary, NC, USA), and statistical significance was tested at level 0.05.

## Results

### Clinicopathologic cohort

Retrospective survey of the archives of autopsied infants with sudden and unexpected death in the medical examiner’s office in San Diego County revealed 227 deaths between 1991 and 2012. Of these cases, 69 % (157/227), ranging in postnatal age from 2 to 358 days (mean 4.2 ± 2.7 months), had at least one technically satisfactory section of the hippocampus for microscopic review. Four infants were excluded from the study due to the presence of gross brain and/or somatic malformations, yielding a total of 153 infant deaths for analysis.

Of the study cohort, 74.5 % (114/153) were unexplained and 25.5 % (39/153) were explained (controls) after a complete autopsy and scene investigation. No infants in either group had known neurological disorders, witnessed seizures (including terminally), nor any gross abnormalities of the hippocampus or temporal lobe. The causes of death in the explained non-suffocation group (*n* = 24) included homicide by head and/or somatic trauma (*n* = 19) or drug overdose (*n* = 1), pulmonary infection (*n* = 2), possible fatty acid oxidation disorder (*n* = 1), and congenital myopathy (*n* = 1). The causes of death in the explained suffocation group (*n* = 15) included accidental drowning (*n* = 7), intentional suffocation with a pillow (*n* = 3), overlaying by sibling (*n* = 2), head entrapment in crib (*n* = 1), birth asphyxia due to complicated delivery (*n* = 1), and acute hypoxia due to massive hemolysis (*n* = 1). The majority of infants in the unexplained group were found in unrecommended sleep environments (69.3 % [79/114]), followed by recommended sleep environments (17.5 % [20/114]), and possible suffocation (10.5 % [12/114]) (Table 2); incomplete scene information prevented subcategorization in three infants. There were no significant differences in incidence of preterm birth, male gender, race, or PMI (Table 2). The unexplained group was significantly younger (15.7 ± 9.6 vs. 25.5 ± 14.4 postnatal weeks, *p* < 0.001) (Table 2). We examined the effect of age (gestational, postnatal, and postconceptional) on all features, including microdysgenetic features, by logistic regression, controlling for the diagnosis/classification. The frequency of 6/42 (14.3 %) features changed significantly with age. Five features decreased with age; one feature, hyper-eosinophilia of the dentate granule cells, increased with age (data not shown). All five features that decreased with age are considered markers of acquired insults, including hypoxia–ischemia, i.e., reactive gliosis of the temporal white matter, and reactive gliosis in CA1–3 and hilus of the hippocampus proper. For each of the six hippocampal features that had a significant effect of age, we controlled for age in the logistic regressions of its frequency in diagnostic subgroups. There was no significant effect of age or PMI on FGCB.

**Table 2:** Clinicopathologic characteristics of the sudden infant death cohort (*n* = 153) adjudicated with asphyxia risk profile

	Explained death	Explained, non-suffocation (ENS)	Explained, suffocation (ES)	Unexplained death	Unexplained, recommended sleep environment (URSE)	Unexplained, unrecommended sleep environment (UUSE)	Unexplained, possible suffocation (UPS)	Insufficient data	*P* value^d^
*n*	39^b^	24^b^	15	114^c^	20	79^c^	12	3	
Postnatal age, weeks	25.5 ± 14.4	23.7 ± 12.1	28.3 ± 17.6	15.7 ± 9.6	20.5 ± 11.5	15.1 ± 8.7	12.8 ± 10.8	13.4 ± 8.2	<0.001^e^
Gestational age, weeks	38.7 ± 2.8	38.1 ± 3.4	39.5 ± 1.1	38.0 ± 3.6	38.2 ± 2.7	38.0 ± 3.7	39.3 ± 1.5	33.3 ± 7.0	0.075
Postconceptional age, weeks	64.5 ± 15.3	62.3 ± 13.2	67.9 ± 18.1	53.8 ± 10.2	58.6 ± 12.0	53.1 ± 9.3	52.0 ± 11.2	46.7 ± 5.6	<0.001^f^
% Preterm birth^a^	18.4 % 7/38	26.1 % 6/23	6.7 % 1/15	21.9 % 25/114	25.0 % 5/20	21.5 % 17/79	8.3 % 1/12	66.7 % 2/3	0.50
% Male	66.7 % 26/39	70.8 % 17/24	60.0 % 9/15	56.1 % 64/114	60.0 % 12/20	54.4 % 43/79	58.3 % 7/12	66.7 % 2/3	0.72
% White	33.3 % 13/39	33.3 % 8/24	33.3 % 5//15	45.5 % 51/112	35.0 % 7/20	46.8 % 36/77	66.7 % 8/12	0.0 % 0/3	0.32^g^
% Hispanic	35.9 % 14/39	37.5 % 9/24	33.3 % 5/15	30.4 % 34/112	45.0 % 9/20	28.6 % 22/77	16.7 % 2/12	33.3 % 1/3	
% African American	20.5 % 8/39	25.0 % 6/24	13.3 % 2/15	13.4 % 15/112	5.0 % 1/20	15.6 % 12/77	16.7 % 2/12	0.0 % 0/3	
% Other race^c^	10.3 % 4/39	4.2 % 1/24	20.0 % 3/15	10.7 % 12/112	15.0 % 3/20	9.1 % 7/77	0.0 % 0/12	66.7 % 2/3	
Postmortem interval, days	0.99 ± 0.50	0.98 ± 0.58	1.01 ± 0.36	0.87 ± 0.33	0.86 ± 0.26	0.88 ± 0.33	0.83 ± 0.47	0.76 ± 0.35	0.62

Mean ± SD or % *n*/*N*

^a^<37 gestational weeks at birth

^b^Missing data on gestational age and prematurity for 1 infant

^c^Missing data on race for 2 infants

^d^Tests across five subcategories, analysis of variance for age and postmortem interval, Fisher exact test for prematurity, *χ* ^2^ test for sex and race

^e^ES significantly older than URSE, UUSE, and UPS; ENS and URSE significantly older than UUSE and UPS

^f^ES significantly older than URSE, UUSE, and UPS; ENS significantly older than UUSE and UPS

^g^Test for race based on White versus Hispanic versus African American and Explained versus Unexplained deaths, due to data sparseness

### Abnormalities of the DG

**Fig. 1:**
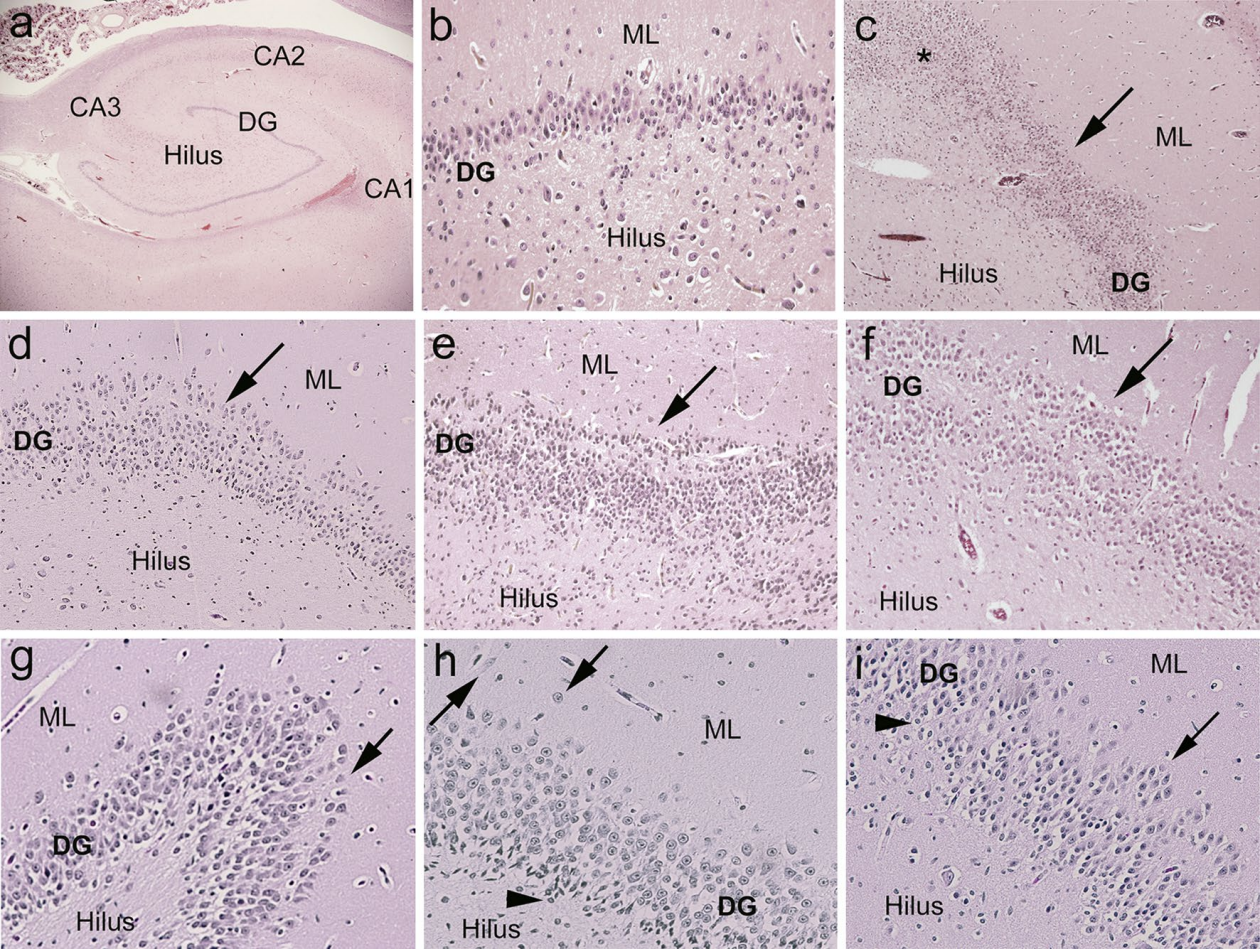
Examples of focal granule cell dispersion in the dentate gyrus (DG) and associated abnormalities in infants with sudden unexplained in death. **a** Normal infant hippocampus with landmarks for reference at its mid-body [level of the lateral geniculate nucleus (not shown)]. The DG forms the shape of a “C” at this level. Hematoxylin and eosin (H&E), ×4. **b** Control DG in a 4-month-old infant with explained cause of death. The DG consists of densely packed granule cells in a linear structure in its straight limbs. H&E, ×20. **c** Hippocampus of an infant with sudden unexplained death with focal granule cell bilamination (*arrow*) in the DG. H&E, ×4. **d** Hippocampus of a second infant with sudden unexplained death with focal granule cell bilamination (*arrow*) in the DG. H&E, ×10. **e** Hippocampus of a third infant with sudden unexplained death with focal granule cell bilamination (*arrow*) in the DG. H&E, ×10. **f** Hippocampus of a fourth infant with sudden unexplained death with focal granule cell bilamination (*arrow*) in the DG. H&E, ×10. **g** Granule cell duplication at the bend (hook) of the DG (*arrow*), associated with FGCB along the straight limb, in an infant with sudden unexplained death. H&E, ×20. **h** There are single, ectopic granule cells in the molecular layer (*arrowheads*). In the DG, there are immature cells, suggestive of immature neurons (Fig. 2), present in the subgranular layer. H&E, ×20. **i** There are immature cells in the DG (*arrowheads*), as well as clusters of granule cells in the molecular layer (*arrow*). H&E, ×20. *DG* dentate gyrus, *ML* molecular layer

**Fig. 2:**
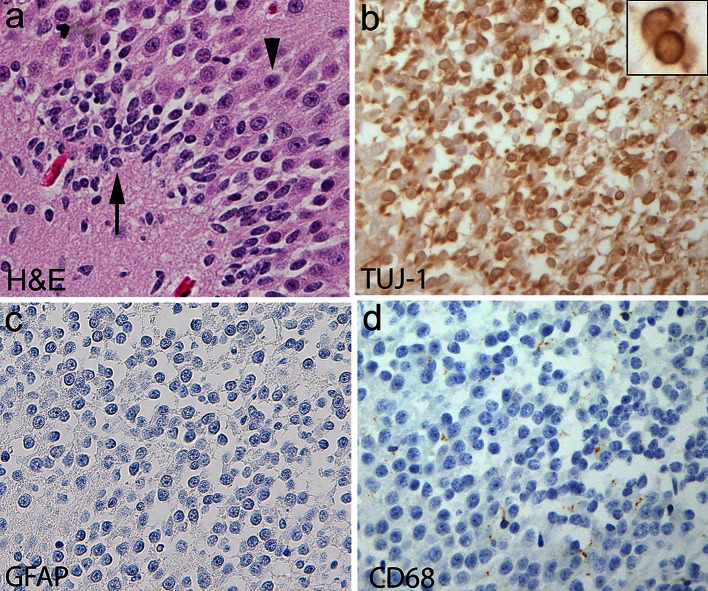
Clusters of immature cells in the DG of a human infant with sudden unexplained death. **a** Histology of immature cells in the deep subgranular layer of the DG (*long arrow*) with small, dark nuclei and negligible cytoplasm compared to mature cells in the upper layers with large nuclei and cytoplasmic differentiation (*arrowhead*). H&E, ×40. **b** The immature cells express cytoplasmic Tuj1, a marker of immature cells of the neuronal cell lineage. The *insert* demonstrates the high nuclear to cytoplasmic ratio of these immature cells, and positive Tuj1 immunostaining within the scant cytoplasm. These cells are not reactive inflammatory cells, as demonstrated by negative immunostaining for GFAP (reactive astrocytes) (**c**), or for CD68 (activated microglia) (**d**). ×40. *DG* dentate gyrus, *GFAP* glial fibrillary acidic protein

**Fig. 3:**
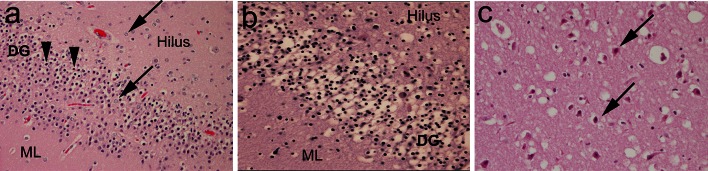
Features of acquired injury, consistent with hypoxia–ischemia. **a** Focal granule cell bilamination associated with hypoxic–ischemic changes (FGCB-HI) in the DG, with a separated line of granule cells (*arrows*) adjacent to the molecular layer, associated with pyknotic neurons and vacuolation (*arrowheads*). H&E, x10. **b** Neuronal necrosis (pyknotic nuclei, shrunken cytoplasm) with vacuolation (edema), consistent with acute hypoxic ischemic injury, in the DG of an infant with explained death. H&E, x20. **c** Acutely necrotic pyramidal neurons in CA1 with hyper-eosinophilic cytoplasm (*arrows*), associated with vacuolation of the neuropil. H&E, x20. *DG* dentate gyrus, *ML* molecular layer

Focal granule cell bilamination was present in 41.2 % (47/114) of the unexplained group compared to 7.7 % (3/39) in the explained (*p* < 0.001) (Fig. 1; Table 3). This lesion was recognized at anterior, mid, and posterior sections of the hippocampus in the unexplained category (data now shown). The frequency of clusters of immature cells in subgranular layer of the DG was also significantly increased in the unexplained group (53.5 % [61/114]) versus 10.3 % (4/39) in the explained group (*p* < 0.001) (Fig. 2; Table 3). Immunocytochemical analysis indicated that these immature cells expressed Tuj1, a marker of early neuronal differentiation (Fig. 2) [42]; these cells did not express immunomarkers of reactive astrocytes or activated microglia (Fig. 2). We identified four features that were significantly associated with the presence of FGCB compared to its absence: (1) bilamination in the bend of C-shaped DG (Fig. 1); (2) clusters of immature cells in the subgranular layer of the DG (Fig. 2); (3) single ectopic granule cells in the dentate molecular layer; and (4) clusters of ectopic granule cells also in the molecular layer (Fig. 1; Table 4). There was no effect of increasing postconceptional age upon the frequency of FGCB, single/clusters of ectopic granule cells, or immature cells in the subgranular layer of the DG. Of the 3/39 (7.7 %) of the explained cases with FGCB, two were subclassified as homicides (one due to blunt head trauma, the other due to intentional smothering), and the third as accidental asphyxia due to wedging in the crib.

**Table 3:** Hippocampal features in the San Diego cohort total *n* = 153 in the unexplained group compared to the explained (control) group

Feature	Explained total *N* = 39	Unexplained total *N* = 114	*p* value^a^
Dentate gyrus features			
Focal granule cell bilamination	3 7.7 %	47 41.2 %	<0.001
Clusters of immature cells in subgranular layer	4 10.3 %	61 53.5 %	<0.001
Focal bilamination at the bend of DG	8 20.5 %	28 24.6 %	0.77^b^
Focal split	2 5.1 %	3 2.6 %	0.60
Focal GC depopulation without HI changes	5 12.8 %	11 9.6 %	0.56
Hyperconvolution	22 56.4 %	63 55.3 %	1.00
Single ectopic granule cells in molecular layer of DG	13 33.3 %	66 57.9 %	0.01
Clusters of granule cells in molecular layer	12 30.8 %	43 37.7 %	0.56
Edema (vacuolation)	18 46.2 %	31 27.2 %	0.05
“Caterpillar” shape of DG	4 10.3 %	15 13.2 %	0.76^b^
Excess medial folds of DG	16 41.0 %	46 40.4 %	1.00
Extra loop of DG	6 15.4 %	11 9.6 %	0.38
Irregular configuration of DG	12 30.8 %	44 38.6 %	0.44
Acquired features in hippocampus/temporal lobe			
Acute or chronic HI without focal granule cell bilamination	25 64.1 %	24 21.1 %	<0.001
Focal granule cell bilamination with hypoxic ischemic changes	6 15.4 %	16 14.0 %	0.80
Clusters of immature/rod cells with HI changes	18 46.2 %	32 28.1 %	0.05
Microhemorrhage in the DG/Ammon’s horn	5 12.8 %	17 14.9 %	1.00
White matter gliosis in temporal lobe	4 10.3 %	17 14.9 %	0.84^c^
Neuronal loss in CA1	6 15.4 %	5 4.4 %	0.03
Gliosis in CA1	4 10.3 %	12 10.5 %	0.41^c^
Neuronal loss in CA2	1 2.6 %	1 0.9 %	0.45
Gliosis in CA2	3 7.7 %	7 6.1 %	0.34^c^
Neuronal loss in CA3	3 7.7 %	1 0.9 %	0.05
Gliosis in CA3	5 12.8 %	5 4.4 %	0.009^c^
Neuronal Loss in hilus	3 7.7 %	0 0.0 %	0.02
Gliosis in hilus	12 30.8 %	42 36.8 %	0.42^c^
Neuronal loss in entorhinal cortex	0 0.0 %	0 0.0 %	1.00
Gliosis in entorhinal cortex	2 5.1 %	0 0.0 %	0.06
Neuronal loss in subiculum	1 2.6 %	0 0.0 %	0.25
Gliosis in subiculum	4 10.3 %	1 0.9 %	0.02
Focal GC loss with HI changes	7 17.9 %	4 3.5 %	0.006
Hyper-eosinophilic neurons in CA1, CA2, CA3, or hilum	18 46.2 %	24 21.1 %	0.004
Hyper-eosinophilic neurons in DG	19 48.7 %	30 26.3 %	0.20^d^
Microdysgenetic features of hippocampus/temporal lobe			
Heterotopia	3 7.7 %	11 9.6 %	1.00
Vertical cortex in temporal gyrus	5 12.8 %	30 26.3 %	0.12
Neuronal clusters in temporal cortex	1 2.6 %	7 6.1 %	0.68
Neuronal cytomegaly in the temporal cortex	1 2.6 %	4 3.5 %	1.00
Excessive white matter neurons in temporal lobe	7 17.9 %	27 23.7 %	0.59^b^
Fused gyri	0 0.0 %	4 3.5 %	0.57
Anomalous formation of entorhinal cortex	0 0.0 %	7 6.1 %	0.19
Ectopic sites of pyramidal cells	0 0.0 %	4 3.5 %	0.96^b^

*GC* granule cells of dentate gyrus, *HI* hypoxia ischemia

^a^Fisher exact test, except as noted

^b^Logistic regression adjusted for postmortem interval (PMI), since the hippocampal feature significantly decreases with PMI

^c^Logistic regression adjusted for postconceptional age, since the hippocampal feature significantly decreases with age

^d^Logistic regression adjusted for postconceptional age, since the hippocampal feature significantly increases with age. See Table 1S (Supplementary data on-line) for definition of the features

**Table 4:** Hippocampal features significantly associated with the presence or absence of focal granule cell bilamination (GCB)

Hippocampal feature	Focal GCB present	Focal GCB absent	*p* value*
Increased frequency with focal GCB present			
Bilamination at bend of DG	48.0 % 24/50	11.7 % 12/103	<0.001
Clusters of immature cells in subgranular zone of DG	80.0 % 40/50	24.3 % 25/103	<0.001
Single ectopic GC in molecular layer of DG	64.0 % 32/50	45.6 % 47/103	0.04
Clusters of ectopic GC in molecular layer of DG	54.0 % 27/50	27.2 % 28/103	0.002
Focal depopulation of GC without associated changes of HI	18.0 % 9/50	6.8 % 7/103	0.05
Excess medial folds in DG	64 % 32/50	29.1 % 30/103	<0.001
Increased frequency with focal GCB absent			
HI change without focal GCB	0.0 % 0/50	47.6 % 49/103	<0.001
Clusters of immature neurons/rod cells with HI changes	0.0 % 0/50	48.5 % 50/103	<0.001
Gliosis CA1	0.0 % 0/50	15.5 % 16/103	0.001
Gliosis CA2	0.0 % 0/50	9.7 % 10/103	0.03
Gliosis CA3	0.0 % 0/50	9.7 % 10/103	0.03
Gliosis CA4	20.0 % 10/50	42.7 % 44/103	0.007
Focal loss of GC in DG with HI changes	0.0 % 0/50	10.7 % 11/103	0.02
Acute neuronal necrosis with hyper-eosinophilia in CA1–3 and hilus	0.0 % 0/50	40.8 % 42/103	<0.001
Acute neuronal necrosis with hyper-eosinophilia in DG	2.0 % 1/50	46.6 % 48/103	<0.001
Edema of DG	8.0 % 4/50	43.7 % 45/103	<0.001

*DG* dentate gyrus, *GCB* granule cell bilamination, *GC* granule cells, *HI* hypoxia–ischemia

* Fisher Exact test

**Fig. 4:**
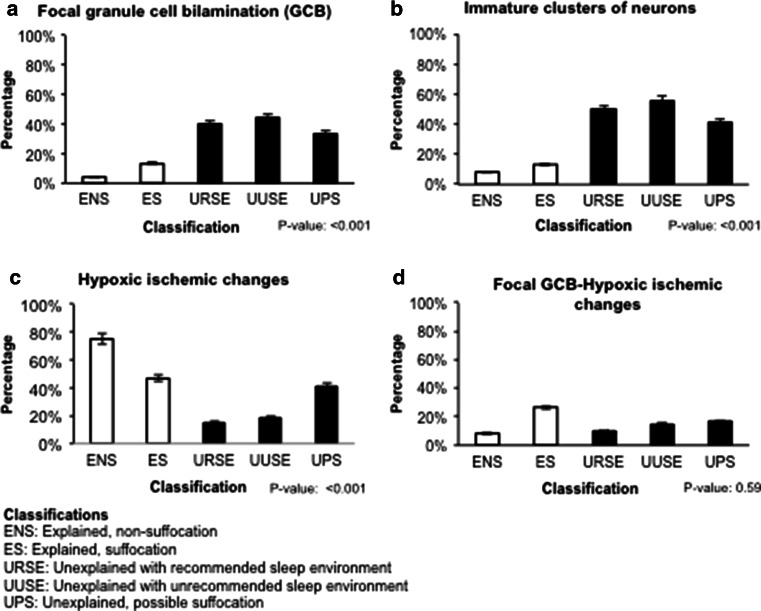
Frequency of the following features in the unexplained (*black*) and explained (*white*) groups and their subcategories. **a** Focal granule cell bilamination (FGCB). **b** Immature clusters of neurons in the subgranular layer of the dentate gyrus. **c** Hypoxic changes in the dentate gyrus and/or Ammon’s horn of the hippocampus, with FGCB. **d** FGCB associated with hypoxic–ischemic changes. *P* value is from a Fisher exact test across the five subcategories

In the unexplained group, there was no significant increase in the frequency in the DG and temporal cortex/white matter of acquired features indicative of acute or chronic hypoxic–ischemic injury (Fig. 3; Tables 3, 4). Yet, 64.1 % (25/39) of the explained group demonstrated acquired features compared to 21.1 % (24/114) of the unexplained group (*p* < 0.001) (Fig. 3; Table 4). As postulated a priori, the frequency of FGCB-HI was not significantly different between the explained (15.4 % [6/39]) and unexplained (14.0 % [16/114]) groups (*p* = 0.80), and this lesion was distributed across all subcategories (data not shown). The presence of FGCB-HI was significantly associated with features indicative of acute and/or chronic injury *outside* of the DG, whereas these same features were not associated with FGCB without dentate HI changes (Fig. 4; Table 4). Thus, the morphological profile of linked features differed significantly between FGCB and FGCB-HI, indicating distinct entities, with FGCB almost exclusively found in the unexplained group.

According to the classification schema of the San Diego medical examiner system, 67.9 % (104/153) were unexplained, and 32 % (49/153) were explained deaths. Focal granule cell bilamination was present in 41.4 % (43/104) of the unexplained (combined SIDS and undetermined categories) compared to 14.3 % (7/49) in the explained (*p* < 0.001). The frequency of FGCB was significantly higher in the SIDS group (43.0 % [37/86]) (without undetermined group) compared to the explained group (14.3 % [7/49]) (*p* < 0.001). In the explained category, homicide was the leading subcategory (50.0 % [24/49), followed by known natural causes (20.4 % [10/49]), accidental suffocation (16.3 % [8/49]), and accidental trauma (drowning in bathtub, 14.3 % [7/49]).

### Microdysgenesis in the temporal lobe

**Fig. 5:**
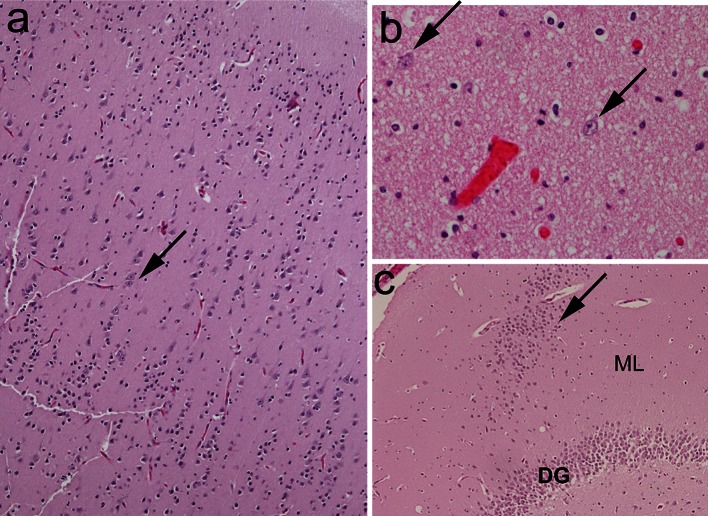
Examples of microdysgenetic features of the hippocampus and temporal lobe assessed in the present study. **a** Vertical cortex with linear columns of neurons (>8 row) (*arrow*) in the temporal cortex. H&E, ×4. **b** Single interstitial neurons (*arrows*) in the white matter of the temporal lobe. H&E, ×20. **c** Heterotopia (*arrow*), comprised of a collection of misplaced granule cells, in the molecular layer. H&E, ×4

There was no significant difference in the frequency of the microdysgenetic features adjusted for age between the explained and unexplained categories (Table 3). Importantly, we observed these features in the infant brains of this entire cohort across a spectrum of causes of death. In the explained category (*n* = 39), considered the “normative” infant population, the frequency of microdysgenetic features analyzed was: excessive white matter neurons (Fig. 5), 17.9 %; vertical cortex (Fig. 5), 12.8 %; heterotopia (in the hippocampus proper or temporal white matter) (Fig. 5), 7.7 %; neuronal clusters in the temporal cortex, 2.6 %; neuronal cytomegaly, 2.6 %; and ectopic pyramidal neurons, 0 % (Table 4).

### Small blood vessel morphology in the hippocampus

**Fig. 6:**
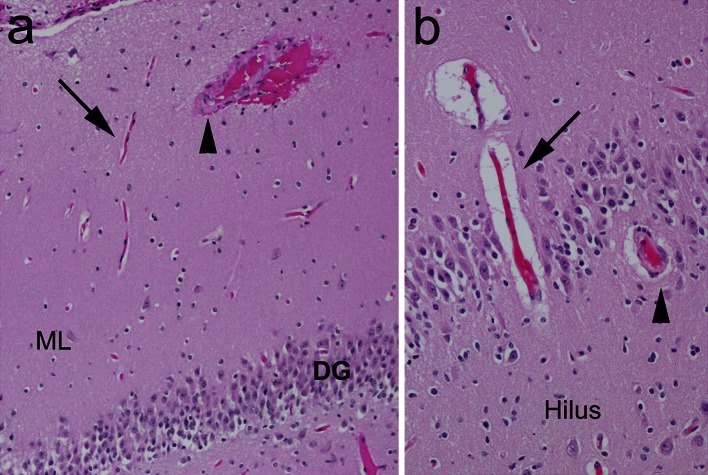
Examples of thick walled blood vessels compared to thin walled blood vessels in the hippocampus proper. **a** Medium-sized artery with thick walls (*arrowhead*) compared to thin walled capillary (*arrow*) in the molecular layer. H&E, ×10. **b** Thick walled vessel (*arrow*) compared to thin walled vessel in the dentate gyrus. H&E, ×20. *DG* dentate gyrus, *ML* molecular layer

There was no significant difference in the frequency of thick walled blood vessels between the explained and unexplained groups. Such vessels were present in 43.6 % of the hilus, 51.3 % of the granule cell layer, and 10.3 % of the molecular layer of the DG in infants with explained causes of death (Fig. 6). The frequency of these blood vessels did not change with age over the first year of life (data not shown). Moreover, the presence of thick walled blood vessels did not correlate with acquired features consistent with hypoxia–ischemia, or with the cluster of features associated with FGCB in explained or unexplained categories.

### Clinical phenotype of unexplained cases with FGCB

Few clinical variables distinguished the unexplained group with FGCB (41.2 % [47/114]) from the unexplained group without FGCB (58.8 % [67/114]). The incidence of prematurity (gestational age at birth <37 weeks) was significantly decreased in the unexplained group with FGCB (10.6 % [5/47]) compared to the unexplained group without FGCB (29.9 % [20/67]) (*p* = 0.015) (Table 5). There were no significant differences in postnatal age, race, gender, or presence of pulmonary edema or intrathoracic petechiae at autopsy.

**Table 5:** Clinical features of unexplained group with focal granule cell bilamination (FGCB) compared to the unexplained group without FGCB

Clinical feature	Unexplained with focal GCB *n* = 47	Unexplained without focal GCB *n* = 67	*p* value^a^
Postnatal age, weeks	15.0 ± 7.8 (2.7–41.0)	16.3 ± 10.7 (0.4–51.1)	0.46
Gestational age, weeks	38.8 ± 2.3 (30–41)	37.5 ± 4.2 (25–42)	0.04
Postconceptional age, weeks	53.7 ± 8.3 (41.9–81.0)	53.8 ± 11.4 (37.0–91.1)	0.99
Male sex	28/47 (59.6 %)	36/67 (53.7 %)	0.54
Premature birth^b^	5/47 (10.6 %)	20/67 (29.9 %)	0.02
Race			0.13^c^
White	19/47 (40.4 %)	32/67 (47.8 %)	
African American	10/47 (21.3 %)	5/67 (7.5 %)	
Hispanic	15/47 (31.9 %)	19/67 (28.4 %)	
Other/Unknown	3/47 (6.4 %)	11/67 (16.4 %)	
Prone sleep position at discovery	21/39 (53.8 %)	25/57 (43.9 %)	0.63^d^
Co-sleeping	13/47 (27.7 %)	26/65 (40.0 %)	0.18
Minor illness around time of death	12/47 (25.5 %)	13/64 (20.3 %)	0.52
Intrathoracic petechiae	36/47 (76.6 %)	57/67 (85.1 %)	0.25
Pulmonary edema	16/47 (34.0 %)	19/67 (28.4 %)	0.52

*NS* not significant

^a^ *t* test for age, *χ* ^2^ test for all others

^b^Less than 37 gestational weeks at birth

^c^Comparison of White versus African American versus Hispanic

^d^Comparison of prone versus supine versus side

## Discussion

The major finding of this study is that 41.2 % of infants with sudden unexplained death (compared to 7.7 % of explained deaths) demonstrate granule cell dispersion in the dentate gyrus of the hippocampus characterized by FGCB. This finding suggests that FGCB may be a morphological marker of an impaired forebrain/limbic network that increases the risk of sudden infant death due to instability of modulation of brainstem cardiorespiratory-related nuclei, or to a subclinical seizure in an infant with a predisposition to epilepsy, which had not yet manifested as a clinical seizure. We propose that this morphological marker “identifies” a vulnerable infant at risk for sudden death during a critical developmental period when the infant meets an exogenous stressor, i.e., the vulnerable infant of the triple-risk model for SIDS [12]. The hippocampus is interconnected with other forebrain loci in the limbic network (e.g., amygdala, insula, hypothalamus), as well as brainstem sites that directly mediate autonomic function and respiration [16, 46, 52, 53, 61]. A major characteristic of the limbic network is its striking susceptibility to seizure generation and propagation, and seizure discharges in temporal lobe epilepsy in particular precipitate serious cardiorespiratory events, e.g., apnea and bradycardia [16, 53]. Autonomic seizures are a consideration in sudden infant death since recurrent episodes of apnea, reported in infants who subsequently die of SIDS [47], may be the sole manifestation of seizures (without movement abnormalities) in infants with temporal lobe pathology [36]. The association of seizures (and sudden death) linked to limbic/hippocampal pathology with sleep periods suggests that the sleep state in some way lowers the threshold for epileptogenesis in the limbic system. In human neuropathology, dispersion of granule cells is a virtually unique feature of temporal lobe epilepsy [1, 2, 5, 17]. Animal models with defective granule cell organization also demonstrate clinical seizures, further implicating abnormal DG morphology in epileptogenesis [31, 42, 43, 48].

The cause(s) of the FGCB in the unexplained subset in this study is unknown. In temporal lobe epilepsy, granule cell dispersion is postulated to be either a primary developmental defect, or secondary to seizures themselves [1, 2, 5, 17]. We suggest that the FGCB in unexplained infant deaths is a developmental defect in neuronal proliferation, migration, and/or cell survival in the DG. The strong association of FGCB with other features of maldevelopment in the dentate gyrus, e.g., excessive single or clustered ectopic granule cells in the dentate molecular layer and hilus, and clusters of immature cells in its subgranular layer, the zone of granule cell neurogenesis, supports the developmental conjecture, as well as the lack of acquired inflammation, i.e., reactive astrocytes and activated microglia.

Granule cell production occurs in the human DG postnatally, with stabilization within the first 3 years [34]. Despite these dynamic changes, granule cell dispersion has not been observed in normal dentate development; rather, its presence is considered pathologic [1, 2, 5, 14, 17, 20]. In this study, the immature cells, labeled by Tuj1, in excessive clusters in the subgranular layer are potentially “stalled” in the subgranular layer due to impaired migration, or alternatively, “accumulate” in the subgranular layer due to an abnormally prolonged cell survival, a mechanism suggested in the PET1 knockout mouse with impaired 5-HT cell development [7].

Important support for this being a developmental lesion is that FGCB did not correlate with features of acquired injury, e.g., neuronal necrosis or loss, and/or gliosis in the DG and/or Ammon’s horn, features consistent with hypoxic–ischemic injury, or with Ammon’s horn sclerosis (AHS) associated with temporal lobe epilepsy [1, 2, 5, 17]. Cases with FGCB-HI may reflect neurogenesis stimulated by HI, as reported in experimental models [4, 29], and thus FBCB-HI is not a feature specific to either group. Thick walled small blood vessels, a feature consistent with a reaction to hypoxia–ischemia [30], do not appear more frequently in the hippocampus in association with neuronal and/or gliotic features of hypoxia–ischemia in either the explained or unexplained categories.

The presence of FGCB without gliosis in infants suggests that this lesion may be the precursor to AHS. Dentate maldevelopment has been postulated to be an abnormal tissue focus that triggers seizures or generates cardiorespiratory instability (independent of seizures). Over time, survivors with this abnormality may develop seizures with recurrent hypoxia–ischemia, excitotoxicity, and/or secondary neuronal loss and gliosis, i.e., Ammon’s horn sclerosis (AHS). Historically, this hypothesis was supported only by the rare reports of granule cell dispersion without AHS in infants [14]. We report it now in 47 unexplained infant deaths and 3 explained infant deaths, and we speculate that infants with FGCB who survive infancy may potentially develop temporal lobe epilepsy and AHS in later life.

The lack of an association of FGCB with microdysgenesis in the temporal lobe in the unexplained deaths is important relative to the potential developmental nature of the dentate lesion. Historically, microdysgenetic alterations have been reported in the temporal lobes of patients with AHS, and are considered to be developmental in origin [1, 2, 5, 7, 17]. Microdysgenesis, however, has also been observed in non-epileptic brains, albeit according to poorly defined criteria, and may reflect normal variation [11, 21]. Therefore, the granule cell dispersion of AHS may be secondary to seizures, an idea supported by the recognition that the mature brain contains stem cells that could be involved in this epilepsy-induced neurogenesis [2]; the brain’s reaction to seizures may create a common pathology that is unrelated to the etiology of the seizures [2]. In the present study, we found that microdysgenetic features are present in 0–17.9 % of normative (explained, seizure-free) infant brains (*n* = 39), depending upon the feature. None of these features change with age across the first postnatal year. Microdysgenetic features were not increased in frequency in the unexplained group compared to the explained group, despite the significantly increased frequency of FGCB in the unexplained group. If microdysgenesis reflects primary developmental abnormalities, then the lack of an increased frequency in the unexplained group with FGCB suggests that maldevelopment is restricted to the dentate gyrus.

In regards to 5-HT brainstem pathology in SIDS and its possible link to hippocampal defects, 5-HT is released from nuclei in the rostral raphe in the caudal midbrain and upper pons to play a trophic role neurogenesis, migration, and neuronal survival in the DG in early development [8, 13, 18, 57]. Moreover, 5-HT from the rostral raphe helps regulate neurogenesis in the DG throughout life. Serotonin dysfunction is implicated in the seizure pathogenesis [57], and hippocampal abnormalities of 5-HT_1A_ receptor binding have been reported in temporal lobe epilepsy [3]. Thus, the underlying vulnerability in the infant at risk for sudden death may reflect a 5-HT brainstem disorder with deficient projections from the rostral raphe to the hippocampus, or alternatively, brainstem and hippocampus disorders independent from one another. Future research is needed to determine the role of brainstem 5-HT in the dentate disorganization reported here.

Importantly, the dentate abnormalities in this study were present in the unexplained group infants with both recommended and unrecommended sleep environments, and with and without possible suffocation. In the last two decades, reductions in infant mortality have been attributed to modifying risks in the infant’s sleep environment, e.g., reducing prone sleep and bed sharing [49, 54, 56]. In parallel, there has been a growing trend to label the cause of deaths with sleep-related risk factors not as “SIDS”, but rather, as “sudden unexpected infant death” (SUID), “undetermined”, “unintentional suffocation”, or “positional asphyxia” in otherwise normal infants [33, 56]. Yet, the triple-risk model proposed by us suggests that at least some SIDS infants, and/or infants with unexplained deaths with sleep-related risk factors, have an underlying vulnerability (disease) that puts them at risk for sudden death when challenged by an environmental stressor, e.g., asphyxia, in the critical developmental period [12]. We further postulate that the infant’s vulnerability is unmasked by sleep combined with the sleep-related stressor by mechanisms yet to be discovered. Our hippocampal findings in the unexplained deaths in unrecommended sleep positions suggest that accidental asphyxia in an otherwise normal infant does not necessarily account for all sudden death in these sleep environments. While asphyxia may trigger acute dysfunction in the abnormal dentate gyrus and an autonomic seizure or cardiorespiratory instability, the presence of the dentate abnormality in infants without environmental risk factors suggests that factors other than asphyxia precipitate death in compromised infants.

Potential limitations of this study relate to the use of archival autopsy materials. First, the majority of cases had only one section of the hippocampus available for review, thus it is possible that the frequency of FGCB is underestimated in both the explained and unexplained groups. Second, an unavoidable limitation is the use of control autopsy infants with acute disorders, e.g., infection, whose influence upon DG structure is currently unknown. Indeed, we found that FGCB was present in 7.7 % (3/39) of infants dying of explained causes but without a history of seizures and/or somatic/brain malformations, suggesting that FGCB is not specific to unexplained death, but rather occurs in the unexplained category with significantly increased frequency. Until the etiology and pathogenesis of FGCB are discovered, the basis of its overlap in explained and unexplained infants is unknown. A third potential limitation in this study of the hippocampus is its retrospective nature in which only archival sections at non-standardized levels were available, and wide-scale immunocytochemical studies were not feasible. Analysis of “uneven cuts” of hippocampus is always a limitation in studies of the human hippocampus, given the changing configuration of this structure anterior to posterior [10, 20]. In this study, however, we examined only sections in which the orientation of the hippocampus could be properly determined. Although this study was hypothesis driven, we emphasize that verification requires future studies in which the entire right and left temporal lobes of forensic infant autopsied cases are examined in serial step sections with precise matching of anterior to posterior levels, and in depth immunocytochemical studies to characterize cell markers of interest. We would argue that our data justifies and mandates such further analyses.

In conclusion, granule cell dispersion is a subtle lesion in an exquisitely restricted site of the hippocampus; yet, pathological changes within it can cause seizures and/or central cardiorespiratory dysfunction, which in turn can be lethal. The finding of FGCB, a distinctive variant of granule cell dispersion, in the hippocampus of infants with sudden unexplained death opens new avenues for research into underlying vulnerabilities of infants to sudden death. Given that granule cell dispersion is a pathologic hallmark of temporal lobe epilepsy, its finding in the brains of infants dying suddenly without explanation raises provocative questions about an underlying anatomic link in limbic-related homeostatic instability between SIDS and temporal lobe seizures associated with SUDC and SUDEP. The descriptive observation of FGCB in sudden infant death is a potentially critical clue towards guiding future neuropathologic studies in human infants, as well as mechanistic testing in developmental animal models with consideration of hippocampal–brainstem interactions. Indeed, the pivotal role of such human neuropathologic descriptions in seeding disease discovery in neurological disease has recently been emphasized [45]. In this regard, further research is needed to determine the relationship between hippocampal and previously reported brainstem pathology in sudden infant death. Importantly, the dentate lesions observed in the unexplained subgroup in this study can be identified by informed microscopic examination, and thus, their determination can be readily incorporated into the forensic investigation of sudden infant death.

## Electronic supplementary material

Below is the link to the electronic supplementary material.

Supplementary material 1 (DOCX 128 kb)
