# Supplementary material for: Dentate gyrus abnormalities in sudden unexplained death in infants: morphological marker of underlying brain vulnerability
Source: Acta Neuropathol. 2014 Nov 25;129(1):65–80. doi: 10.1007/s00401-014-1357-0 (PMC4282685; doi:10.1007/s00401-014-1357-0)
Supplement: Supplementary file 1 — Supplementary material 1 (DOCX 128 kb) [file 401_2014_1357_MOESM1_ESM.docx]

**Supplementary Data**

Table S1. Definitions of the morphologic features in the hippocampus/temporal lobe analyzed in the study.

**A. Dentate Gyrus Features**

1. Focal granule cell bilamination (FGCB) without acute and/or chronic hypoxic- ischemic changes (Figure 1)

There is a linear row of at least 8 ectopic granule cells that is separated from the main dentate gyrus (DG) in the supragranular or subgranular sites. This may be FGCB or complete granule cell bilamination, the latter change not identified in any of the hippocampi of this study. There is an acellular, eosinophilic zone of neuropil between the line of ectopic cells and the main DG. The linear row may be one or more cells thick. This change must be on the “straight” limb and not at the bend of the DG. There may be pyknotic nuclei in the subgranular zone, which may represent apoptosis. This latter finding alone does not warrant the diagnosis of hypoxic ischemic change because it may be part of a cellular disturbance in neurogenesis with apoptosis.

2. Focal granule cell bilamination at the bend of the DG (Figure 1)

There is a linear row of at least 8 ectopic granule cells above the DG at the bend of the C- shaped DG. This linear row is one or more cells thick.

3. Focal split in DG that looks like bilamination

The DG separates into two branches, each branch going forward parallel to each other. This diagnosis is not considered if a blood vessel is present at the origin of the split.

4. Dark, round, immature cells in clusters in the subgranular and deep layers of DG, without associated hypoxic-ischemic (HI) changes (Figure 1)

These clusters are comprised of oval or round cells with delicate chromatin without the eosinophilic cytoplasm of the mature granule cell. They resemble undifferentiated neural/glial progenitor cells (Figure 2). They are usually in clusters in the lower layers of the DG, but occasionally form lines up into the DG, suggesting migration.

5. Single ectopic granule cells in the molecular layer of DG (Figure 1)

Single granule cells are scattered above the supragranular layer and in the molecular layer of the dentate gyrus.

6. Clusters of ectopic granule cells in molecular layer of the DG

Groups of 2-4 granule cells are scattered above the supragranular layer of the dentate gyrus, and can extend out into the molecular layer (>10 microns). The granule cells form circular clusters, not lines as in FGCB.

7. Hyperconvolution of the dentate gyrus

The dentate gyrus is altered from its normal C or oval shape by one or more loops of invaginated or evaginated folds of the full thickness of the DG.

9. Irregularity of DG

The thickness of the granular cell layer of the DG is uneven, varying between being thick and thin, the irregular DG is continuous without gaps of granule cell depopulation. There may or may not be hyperconvolution.

10. Focal granule cell (GC) absence in DG without gliosis

There is a focal absence or decrease in number of granule cells. This change is not associated with gliosis, and may reflect a developmental underpopulation of GC, rather than acquired cell loss.

11. “Caterpillar” appearance of DG

The thickness of the granule cell layer of the DG is altered by a series of regular rounded thickenings. These are on one edge towards the supragranular zone where they create an undulating border. It is a form of an irregular DG.

12. Excessive folds on DG on medial surface of the hippocampus

There are 2 or more folds on the medial surface of the body of the hippocampus abutting the temporal horn of the lateral ventricle.

13. Extra-loop of DG

A loop of dentate gyrus is isolated from main body at any level of the hippocampus remote from the pes.

**B. Acquired Features of the Dentate Gyrus, Ammon’s Horn, and Temporal Cortex/White Matter**

14. FGCB with acute or chronic hypoxic-ischemic (HI) changes (Figure 3)

This is the same as FGCB but associated with acute or chronic hypoxic-ischemic (HI) changes. *Acute HI changes*: Hyper-eosinophilic granule cells, dying (pyknotic) granule cells, karyorrhexis, pyknosis, and/or vacuolation (edema). There are typically also HI changes in Ammon’s horn and temporal cortex. *Chronic HI changes*: Gliosis in DG, alone or with acute changes; loss of DG cells (drop out) and gliosis associated with thinning of DG. There may be chronic changes in AH and/or temporal cortex with gliosis and/or neuronal loss.

15. Hypoxic-ischemic changes only with no FGCB (Figure 3)

There are HI changes of variable degrees without FGCB. HI changes may be acute or chronic (see above): *Acute HI changes*: Hyper-eosinophilic granule cells, dying granule cells, karyorrhexis, pyknosis, and/or vacuolation (edema). There are typically also HI changes in Ammon’s horn and temporal cortex. *Chronic HI changes*: Gliosis in DG, alone or with acute changes; loss of DG cells (drop out) and gliosis associated with thinning of DG. There may be chronic changes in AH and/or temporal cortex with gliosis and/or neuronal loss.

16. Dark round cells in clusters and Rod cells with hypoxic ischemic changes

These are small, round, or rod-shaped cells in the subgranular layer or throughout the dentate gyrus associated with acute and/or chronic HI changes. In this context, they may represent neurogenesis stimulated by hypoxia-ischemia. Rod cells here also suggest activated microglia. These dark round cells and rod cells are distinguished from dying cells because their nuclei are not pyknotic, but delicate and distinct.

17. Microhemorrhage in the DG or Ammon’s horn

There is petechial or microhemorrhage within the granule cell layer or molecular layer in the DG, or within the pyramidal cell or molecular layer of AH at any level.

18. Gliosis in the white matter of the temporal lobe

There are reactive astrocytes with naked, enlarged nuclei without cytoplasm (naked glia) or hypertrophic astrocytes (with enlarged, eosinophilic cytoplasm) in the cingulum, optic radiation, or central white matter of the temporal lobe.

19. Hypereosinophilic neurons in CA1-3 and/or hilus (Figure 3)

There are intensely eosinophilic pyramidal cells in the subdivisions of Ammon’s horn. If these are isolated and rare, this code is not used. The change has to be promeninent and global.

20. Neuronal loss in CA1

There is an obvious drop-out of neurons, leaving spaces in neuropil.

21. Gliosis in CA1

There are increased numbers of reactive astrocytes with naked, enlarged nuclei without cytoplasm (naked glia) or hypertrophic astrocytes.

22. Neuronal loss in CA2

There is an obvious dropout of neurons, leaving spaces in neuropil.

23. Gliosis in CA2

There are increased numbers of reactive astrocytes with naked, enlarged nuclei without cytoplasm (naked glia) or hypertrophic astrocytes.

24. Neuronal loss in CA3 T

Here is an obvious dropout of neurons, leaving spaces in neuropil

25. Gliosis in CA3

There are increased numbers of reactive astrocytes with naked, enlarged nuclei without cytoplasm (naked glia) or hypertrophic astrocytes.

26. Neuronal loss in hilus

There is an obvious dropout of neurons, leaving spaces in neuropil.

27. Gliosis in hilus

There are increased numbers of reactive astrocytes with naked, enlarged nuclei without cytoplasm (naked glia) or hypertrophic astrocytes. In the hilum naked glia may be seen as isolated or paired nuclei underneath the subgranular layer of the dentate gyrus, or they can be observed throughout CA4.

28. Hypereosinophilic neurons in DG

There are large numbers of intensely eosinophilic granule cells in the DG. If they are scattered (isolated) and rare, this code is not used. The change has to be promeninent and global.

29. Focal loss of DG cells with associated HI changes

There is drop out of granule cells, leaving a “space”. There may be an associated gliosis but there are also hypoxic ischemic changes in the hippocampus. There is gliosis in the DG at times as well. This resembles the classic feature of DG cell loss in hypoxia ischemia and/or epilepsy.

30. Vacuolation in the DG (Figure 3)

The granule cells appear separated by vacuoles or clear spaces in the neuropil. It suggests edema.

31. Neuronal loss in entorhinal cortex

There is an obvious dropout of neurons, leaving spaces in neuropil.

32. Gliosis in entorhinal cortex

There are increased numbers of reactive astrocytes with naked, enlarged nuclei without cytoplasm (naked glia) or hypertrophic astrocytes.

33. Neuronal loss in subiculum

There is an obvious dropout of neurons, leaving spaces in neuropil.

34. Gliosis in the subiculum

There are increased numbers of reactive astrocytes with naked, enlarged nuclei without cytoplasm (naked glia) or hypertrophic astrocytes.

**C. Microdysgenetic Features**

35. Vertical cortex in temporal gyri (Figure 5)

The neurons in the temporal cortex are aligned vertically, with cells in columns of >8 cells in line. There is intervening pink neuropil that is acellular between the columns. Columns are vertical and cross all laminae perpendicular to the pial surface; oftentimes the columns are present in the lower layers.

36. Heterotopia (Figure 5)

There are ectopic collections of neurons that may resemble granule cells in morphology. These heterotopia are misplaced in gray or white matter, and may be found near the junction of the alveus and fimbria, or in the alveus itself.

37. Neuronal clusters in temporal cortex

There are two or more touching neurons in AH, parahippocampal gyrus, or temporal cortex.

39. Cytomegaly of neurons in temporal cortex

The size of the neuron is abnormally large, with increased cytoplasm. These are seen in type II focal cortical dysplasia. The neurons should be distinguished from mossy neurons in the hilus.

40. Excessive interstitial neurons in cingulum or temporal white matter (Figure 5)

There are increased numbers of mature neurons (more than 3/hpf) in the white matter of the cingulum or central temporal lobe white matter.

41. Fused gyri in the temporal cortex

In the temporal cortex, there is fusion of the molecular layers of two adjacent gyri with obliteration of the subarachnoid space.

42. Anomaly of formation of the subiculum/entorhinal cortex

This diagnosis includes several anomalies: an unusual orientation of the entorhinal cortex towards the subiculum, acellular patches in the cortex, or duplication of the subiculum.

43. Ectopic sites of pyramidal cells

This abnormality is diagnosed when single pyramidal-like neurons are observed in the molecular layer of the DG or other ectopic sites, such as layer I of the subiculum, or in upper layers in row in parahippocampal gyrus.

**D. Blood Vessel Feature**

44. Thick walled blood vessel (Figure 6)

This feature is present in medium and small arteries, and in capillaires. The walls of the blood vessel appear increased in width due to increased thickness of the media, associated with prominence of the endothelium. The thick walled vessels are identified in the molecular layer, dentate gyrus, and/or hilus. They are not assessed in the leptomeninges or hippocampal fissure. Only one thick walled vessel needs to be identified, although there is a spectrum in number and density.
